# In-silico identification and prioritization of therapeutic targets of asthma

**DOI:** 10.1038/s41598-023-42803-w

**Published:** 2023-09-21

**Authors:** Ishita Mallick, Pradnya Panchal, Smita Kadam, Priyanka Mohite, Jürgen Scheele, Werner Seiz, Amit Agarwal, Om Prakash Sharma

**Affiliations:** 1 Innoplexus Consulting Pvt. Ltd, 7th Floor, Midas Tower, Next to STPI Building, Phase 1, Hinjewadi Rajiv Gandhi Infotech Park, Hinjawadi, Pune, Maharashtra 411057 India; 2 Innoplexus AG, Frankfurter Str. 27, 65760 Eschborn, Germany

**Keywords:** Computational biology and bioinformatics, Drug discovery, Molecular biology, Biomarkers, Health care

## Abstract

Asthma is a “common chronic disorder that affects the lungs causing variable and recurring symptoms like repeated episodes of wheezing, breathlessness, chest tightness and underlying inflammation. The interaction of these features of asthma determines the clinical manifestations and severity of asthma and the response to treatment" [cited from: National Heart, Lung, and Blood Institute. Expert Panel 3 Report. Guidelines for the Diagnosis and Management of Asthma 2007 (EPR-3). Available at: https://www.ncbi.nlm.nih.gov/books/NBK7232/ (accessed on January 3, 2023)]. As per the WHO, 262 million people were affected by asthma in 2019 that leads to 455,000 deaths (https://www.who.int/news-room/fact-sheets/detail/asthma). In this current study, our aim was to evaluate thousands of scientific documents and asthma associated omics datasets to identify the most crucial therapeutic target for experimental validation. We leveraged the proprietary tool Ontosight^®^ Discover to annotate asthma associated genes and proteins. Additionally, we also collected and evaluated asthma related patient datasets through bioinformatics and machine learning based approaches to identify most suitable targets. Identified targets were further evaluated based on the various biological parameters to scrutinize their candidature for the ideal therapeutic target. We identified 7237 molecular targets from published scientific documents, 2932 targets from genomic structured databases and 7690 dysregulated genes from the transcriptomics and 560 targets from genomics mutational analysis. In total, 18,419 targets from all the desperate sources were analyzed and evaluated though our approach to identify most promising targets in asthma. Our study revealed IL-13 as one of the most important targets for asthma with approved drugs on the market currently. TNF, VEGFA and IL-18 were the other top targets identified to be explored for therapeutic benefit in asthma but need further clinical testing. HMOX1, ITGAM, DDX58, SFTPD and ADAM17 were the top novel targets identified for asthma which needs to be validated experimentally.

## Introduction

Asthma is a chronic disease characterized by recurrent and variable symptoms including episodes of wheeze, cough, chest tightness, dyspnea and backed by variable airflow limitation, airway inflammation and airway hyper-responsiveness^1^. The current rationale for asthma pharmacotherapy focuses on reducing the symptoms that result from airway obstruction and inflammation^2^, Inhaled corticosteroids^2,3^, leukotriene modifiers^4,5^ combination inhalers and theophylline are some of the current medications approved for this purpose by the regulatory authorities. Steroidal inhalers help reduce asthma exacerbation when taken regularly, however; all these medications only relieve asthma symptoms and cannot cure the disease as the high minimal inflammation-causing variable airflow obstruction or limitation is still irreversible. Long-term requirements of inhaled corticosteroids create adherence issues which contribute towards a high level of uncontrolled asthmatics. More than 60% of current asthmatics were found to be uncontrolled in the United States alone^6^ and the situation is even worse in LMICs)^7–10^.

Despite the availability of high-quality clinical and preclinical data, a number of promising drugs have failed in the late clinical stages leading to a gap between basic and clinical research output and the patients’ need for better treatments. These facts encourage us to investigate further on the molecular mechanisms and targets for potential and effective therapeutic interventions. With the advancement of computational techniques and big data availability, now it is quite possible to investigate segregated clinical and preclinical data together to identify most potential targets in asthma.

In this current research work, we evaluated thousands of asthma associated molecular targets and patient expression datasets to identify most relevant therapeutic targets for clinical development. We leveraged the proprietary Ontosight^®^ Discover platform (https://ontosight.ai/) (US20200090789A1) which extracts all biological entities from publications, clinical trials, grants, congresses and patents based on contextual search (https://doi.org/10.2174/2666958702101010205). On the other hand, transcriptomics and genomic datasets were retrieved from the Gene Expression Omnibus (GEO) to identify disease-specific differential expression genes using a machine learning approach. Identified targets and their genomic and expression regulations were integrated and analyzed in a research graph to understand various molecular connections in biological networks to identify the most relevant asthma targets.

The targets were further scrutinized based on their biological relevance, asthma-associated pathophysiological processes, small molecule and antibody druggability, genomic mutations, gene and protein expression, molecular interactions and biological functions, novelty, safety, target structure, localization and the target’s biomarker potential in asthma to list the most promising therapeutic targets for asthma. We identified HMOX1, ITGAM, SFTPD and ADAM17 as the top novel targets for asthma which need to be validated experimentally whereas IL-18, TNF and VEGFA as the strongest therapeutic targets to investigate further for clinical benefit.

## Materials and methods

**Figure 1:**
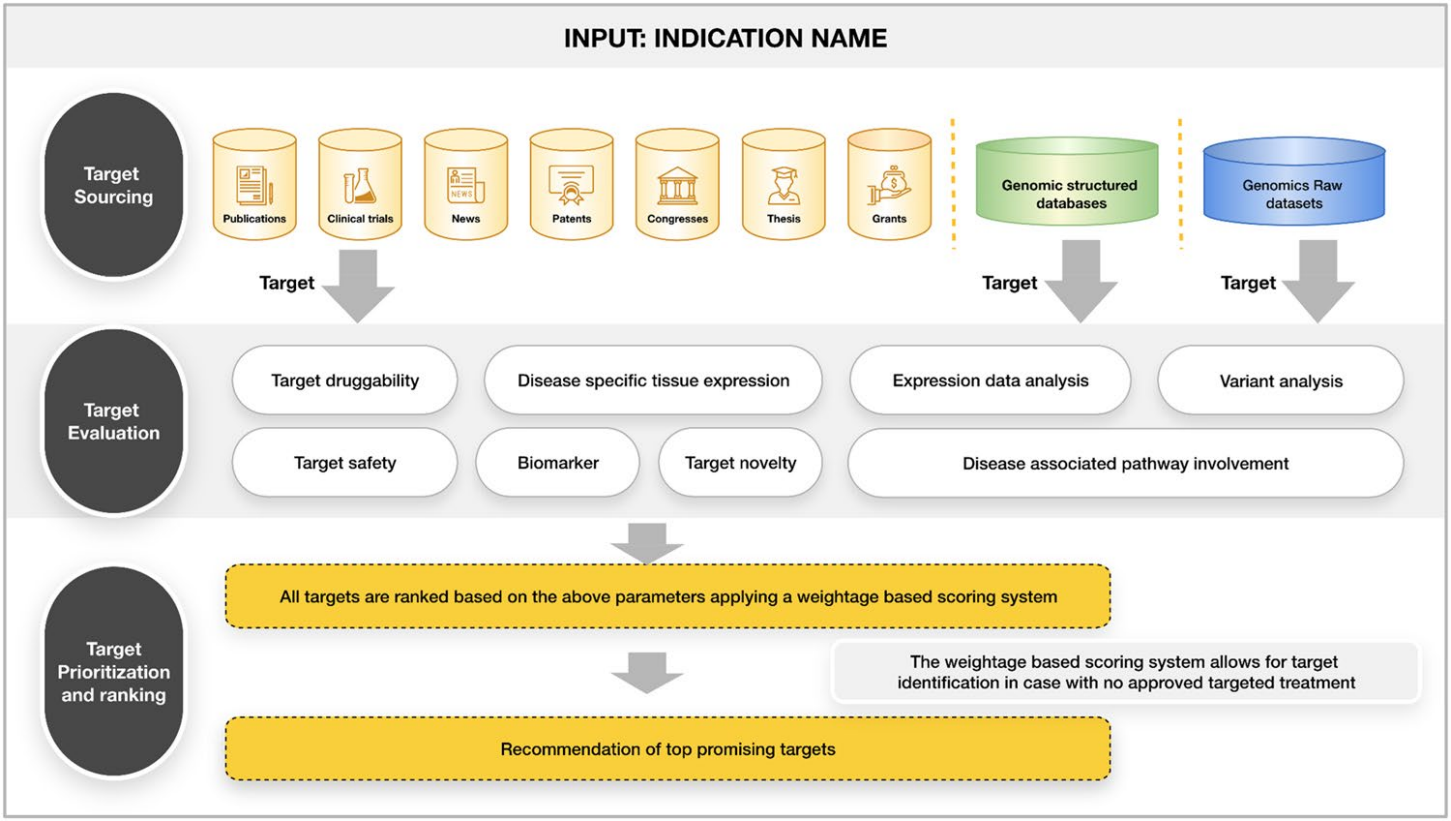
The overall approach for target identification including target identification, evaluation and prioritization.

The approach for target identification consisted of three major stages namely target sourcing followed by target evaluation and then target prioritization as shown in Fig. 1. Here, a very comprehensive approach has been adopted to identify potential asthma-associated targets using Ontosight^®^ Discover. This approach involves identifying proteins from the disease-associated (asthma) biological entities extracted from publications, patents, theses, congresses, grants, and clinical trials. Additionally, publicly available curated databases such as GEO and GWAS, Clinvar, Disgenet and genomics datasets were used to extract and analyze population-based and evidence-based targets associated with asthma.

### Mining unstructured datasets using Ontosight^®^ Discover

Two standard approaches were used to extract an asthma-associated target list. In the first approach, a proprietary tool Ontosight^®^ Discover was used to search thousands of publications, clinical trials, grants, patents, news and congress documents to identify all the relevant and mentioned biological targets. The input query term used here was “asthma” to fetch relevant hits from all mentioned document types. For searching documents, Ontosight^®^ Discover uses the proprietary life science ontology which considers all the pre-validated synonyms for asthma. The target hits from this approach were further filtered based on their relevance score for the selected disease.

**Figure 2:**
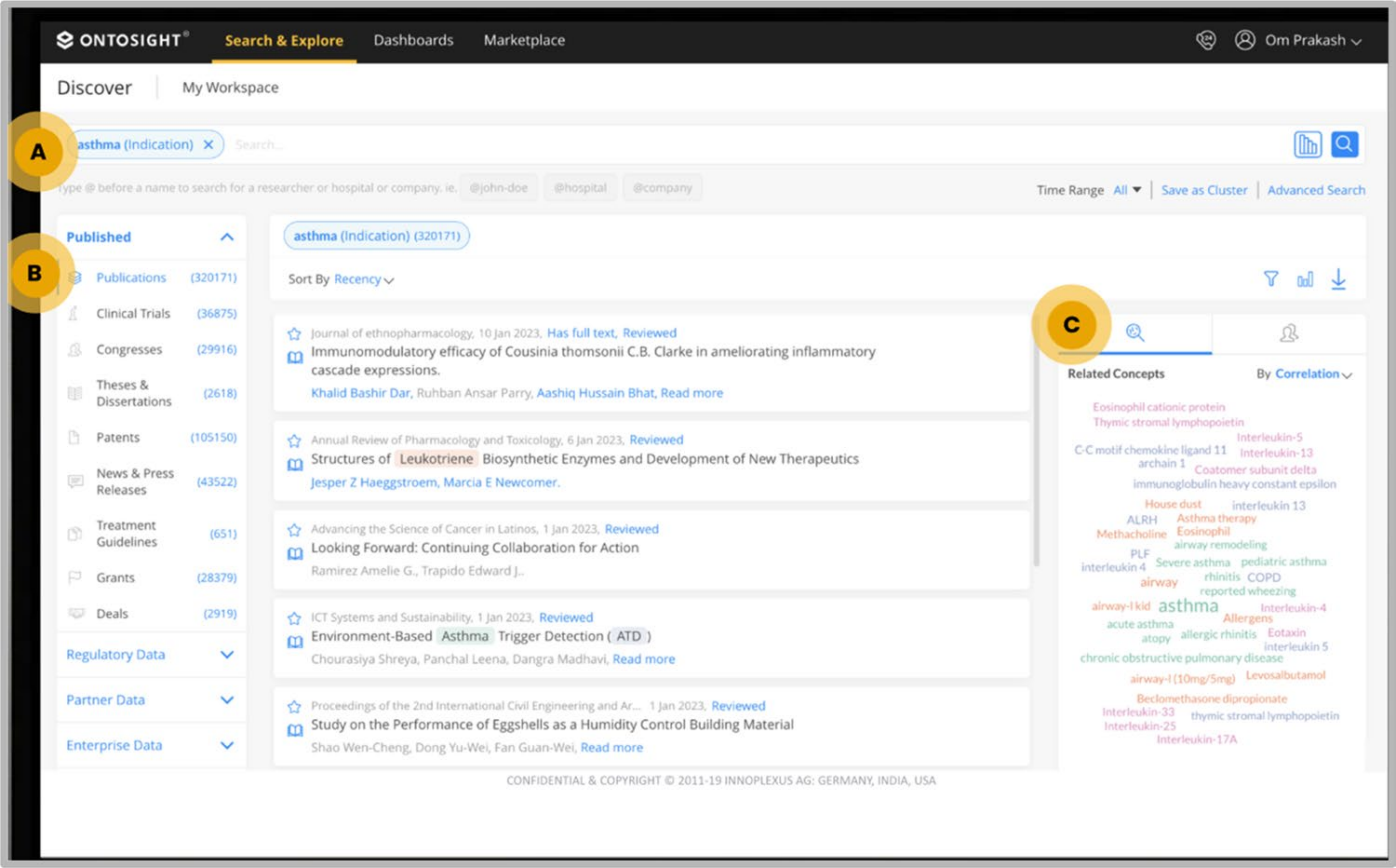
Result page of Ontosight^®^ Discover. Number of documents counts, and relevant targets as shown in this image. Here, (**a**) represents the ontology-based search (**b**) represents the various document types such as Publications, Clinical Trials, Congresses, Theses and dissertations, Patents, News and Press and Grants (**c**) represents various biological terms including target associated with asthma. Here pink color demonstrates proteins, while genes are highlighted in purple color and disease name is highlighted in green color.

This approach of target sourcing involves the mining of targets from the above mentioned 6 document types as shown in below Fig. 2.

### Mining genomics data for potential targets

Genomic datasets help to identify targets which are genetically associated with the pathophysiology of the disease. We did this by two approaches: (a) genomic targets from structured genomic databases and (b) genomic targets from raw patient datasets.

#### Genomic targets from structured genomic databases

We explored curated genomic databases to integrate known and reported targets for asthma. These targets were identified based on the already processed datasets available in the publicly curated databases such as Disgenet (https://www.disgenet.org/), GWAS central (https://www.gwascentral.org/), GWAS catalog(https://www.ebi.ac.uk/gwas/) and ClinVar (https://www.ncbi.nlm.nih.gov/clinvar/). We further considered raw patient expression datasets to identify any other potential targets using genomic expression and mutation analysis.

#### Genomic expression and mutation data analysis for relevant targets

##### Genomics expression analysis

**Figure 3:**
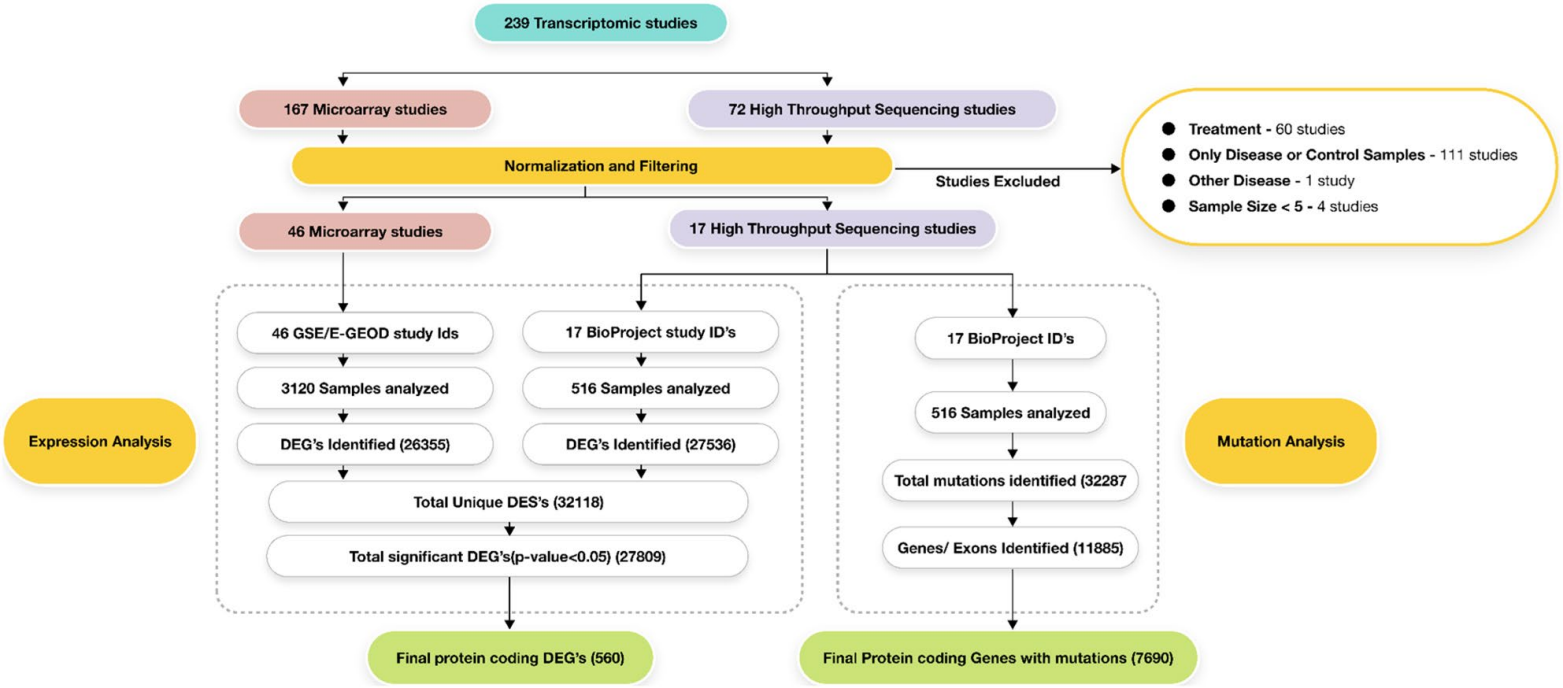
The overall workflow of Genomics analysis.

Genomics expression analysis and genomics variant analysis was performed using the raw sequencing data available on Gene Expression Omnibus (GEO)^11^. The search term “asthma” AND "Homo sapiens"[porgn:__txid9606]” was used to query the number of studies in the GEO database (series under entry type). The search query resulted in 293 hits [as of 6th November 2022] which were asthma associated studies having expression and/or mutation data. In the next step, these 293 studies were filtered based on the *Study Type* to select expression profiling by array (Microarray) and expression profiling by high throughput sequencing (HTS). These filters reduced the study count to 239 which were individually validated before initiating the analysis. Studies with sample count less than 5 (04), studies involving other diseases along with asthma (01), studies with only disease or only control samples (111), studies involving drug treatment (60), studies with knockout or overexpressed genes were discarded from further analysis. These filters helped us arrive at studies that were most relevant to our analysis of identifying only those targets that were differentially expressed when normal versus disease samples were compared (Fig. 3).

These 63 studies were analyzed for differential expression and mutations as follows: The expression data for the downloaded sample files was evaluated using HTS (High throughput sequencing) and microarray. HTS analysis was performed on raw data files and hence multiple normalization and QC steps were followed for the same. On the other hand, microarray analysis required soft QC files which were already normalized and hence did not require additional QC steps unlike HTS data before generating the expression files.

##### Differential gene expression detection and analysis

Data processing and analysis was performed after the relevancy and coverage check. The following steps were followed for evaluating the expression profile of Asthma targets:

### Sample quality check

Implementation of FastQC. The FastQC^12^, was utilized as a tool for quality check. It provided a quality report for raw FASTQ sequencing data, visualizing the statistics and quality of the samples in detail. Standard Adapter and quality removal—The adapter sequences added at the start and end of each of the raw reads were removed as part of sample QC through a process called read trimming. The addition of adapter sequences leads to bulky files with low quality of sequences. These issues were addressed using two trimming tools—FastQC and Cutadapt.

GC percentage for each sample was obtained from the QC Report and samples with GC percentage greater than 60% and less than 40% were listed separately as 50–60% is the recommended GC content. Samples having abnormal GC content were analyzed using GC Bias filter salmon and the rest of the samples were processed through standard parameters. Overrepresented sequences were basically information regarding the sequence contamination according to contamination databases that matched with the sequence present in the sample data. Such sequences with minimum length of ~ 20 bp, if found in the database, were extracted and removed explicitly by using Trim Galore. After the raw data QC, these normalized counts are further analyzed in Deseq2 for calculation of log fold change. After DeSeq2 analysis, a csv file was generated with columns described in Table 1.

**Table 1:** Metadata of deseq2 output (csv) file and their columns name.

#	Column name	Description
1	Gene symbol	Gene symbol based on the Entrez Gene ID
2	Control expression (mean value)	Mean or average of expression values in control samples
3	Disease expression (mean value)	Mean or average of expression values in disease or test samples
4	Log2FC	Log2 fold change between the groups. e.g., value 2 means that the expression has increased fourfold
5	p-value	Wald test p-value
6	p-adj	Benjamini–Hochberg adjusted p-value
7	ABC LogFC	Absolute value of LogFC
8	Gene regulation type	Up/down, to know whether the gene is up regulated or down regulated

#### Genomics mutation analysis

Data collection and normalization for genomics mutation analysis was similar to the genomics expression analysis which is discussed above.

##### Reference genome (hg19) alignment

Spliced Transcripts Alignment to a Reference (STAR) alignment tool was utilized to align our sequence dataset to the reference genome by > 50 mapping speeds^13^**.**

##### Data pre-processing

Data cleanup was done using Picard’s *AddOrReplaceReadGroup* and *MarkDuplicates* functions. *AddOrReplaceReadGroup* enables to replace all the read groups in input sequence with a new read group, while *MarkDuplicates* tool identifies the duplicate sequence present in a given input file (BAM or SAM file).

*Variant calling *We used Genome Analysis Toolkit (GATK); to identify the variants in a given input dataset, which is a standard tool for identifying the SNPs and INDELs in germline DNA and RNAseq data. In the first step of variant calling, SplitNCigarReads was used. CIGAR was used to indicate the match, mismatch, insertions, deletions etc. that were not in the reference. SplitNCigarReads splits and eliminates the reads that contain “Ns” in their CIGAR string. After splitting the read, HaplotypeCaller was used to identify the variants. GATK’s variant filtering guideline was followed to filter the variants where we used two tools—GATK’s SelectVariants and VariantFiltration. First, SelectVariant tools were used to extract only SNPs and INDELs from raw VCF files^14^. The VariantFiltration tool was then used to retain only high-quality SNPs and INDELs. We applied the filters present in Table 2 on raw SNP and INDELs VCF file to remove bad variants from the list.

**Table 2:** Filters applied on raw SNP and INDELS in mutation analysis.

Parameter	Function	Threshold
QuaByDepth (QD)	QD is the variant quality divided by unfiltered depth of non-hom-ref samples	> 2
FisherStrand (FS)	Strand Bias tells us whether the alternate allele was seen more or less often on the forward or reverse strand than the reference allele	< 60
RMSMappingQuality (MQ)	It is the root mean square mapping quality over all the reads at the site	> 40
MappingQualityRankSum Test (MQRankSum)	It compares the mapping qualities of the reads supporting the reference allele and the alternate allele	> − 12.5
ReadPosRankSum Test (ReadPosRankSum)	It compares whether the positions of the reference and alternate alleles are different within the reads	> − 8
StrandOddsRatio (SOR)	Strand Odds is another way to estimate strand bias using a test similar to the symmetric odds ratio test	< 4

*Variant annotation *ENSEMBL’s Variant Effect Predictor (VEP) tool was used to know the functional effect of identified variants on genes^15^.

*Downstream analysis *Variant Annotation generates the list of filtered high quality mutations in Variant Call Format (VCF). Further, we filtered the variants based on the parameters which are listed below

- Biotype should be “Protein Coding”
- Allele Frequency (AF) < 0.01
- Exome Aggregation Consortium (ExAc_AF) < 0.01
- Variant type should not be “Synonymous Variant”
- Impact should be High, Moderate or Low and not only “Modifier”

Loss of function (LOF) analysis was performed only on the variants which had variant type as “Missense variants”. The Sorting Intolerant from Tolerant (SIFT) and Polymorphism Phenotyping V2 (Polyphen-2) status which were present in the filtered VCF file were used to predict the loss of function effect of the variant. If the SIFT status is deleterious and Polyphen-2 status is damaging, then those variants were considered as a Loss of Function variants^16^**.**

### Combined list of targets

The overall target list that was generated from the three sources i.e., unstructured datasets (7237 targets), structured genomic dataset (2932 targets), genomic and variant analysis (8250 targets) were combined together to make a unique list of 12,090 asthma associated targets that were further processed to evaluate their potential in asthma pathophysiology (Table 3).

**Table 3:** Long list of targets identified from structured and unstructured sources. Asthma relevant document count was the total number of documents/studies identified from the given target source and target count was the total number of targets identified from the given number of documents.

Data type		Target source	Asthma relevant documents counts	Target count
Ontosight^®^ Discover (unstructured data)		Scientific publications	329,583	2805
		Clinical trials	13,602	122
		Patent documents	96,840	1366
		Grants	27,699	826
		Congresses	29,597	1007
		News and press	911,605	767
		Theses	2442	344
Genomics data	Genomic database (structured data)	ClinVar database	43,363	14
		Disgenet database	366,315	681
		GWAS central	216,070	1484
		GWAS	176,271	753
	Genomic raw data (from GWAS and Microarray analysis)	Mutation analysis targets	559,063	7690
		Expression analysis targets	46,708	560
		Total	2,819,158	18,419

In the next step, the literature data scoring approach identified the most relevant targets for asthma and removed the least relevant (Table 4). This approach enriched the literature class count for each of these unique 12,090 targets. Targets with a total literature class documents count of less than 20 were excluded from the list as they were considered to be the least relevant targets with mostly non-specific hits.

**Table 4:** Scoring algorithm for asset class score calculation. Parameter score is a sum of the number of citations, the reputation of the journal/Impact factors, date of publications and concept relevancy score using Term frequency (TF)—Inverse document frequency (IDF).

Asset class	Parameters	Parameter level scoring	Class score	Scaled score
Publication	Recency, Impact factor Relevancy (Position of the term in the article)	Sum of all the parameter scores of each document for the relationship	Every parameter has a parameter weightage Class score is calculated by considering the percentage of weightage for each parameter	Asset class score of each asset class is then scaled between 0 to 1 range
Clinical Trials	Clinical trial status, Phase and disease relevance	Sum of all the parameter scores of each document for the relationship		
Patents	Agency which holds the patent recency and relevancy	Sum of all the parameter scores of each document for the relationship		
Grants/Congress/News	Recency and relevancy	Sum of all the parameter scores of each document for the relationship		

**Figure 4:**
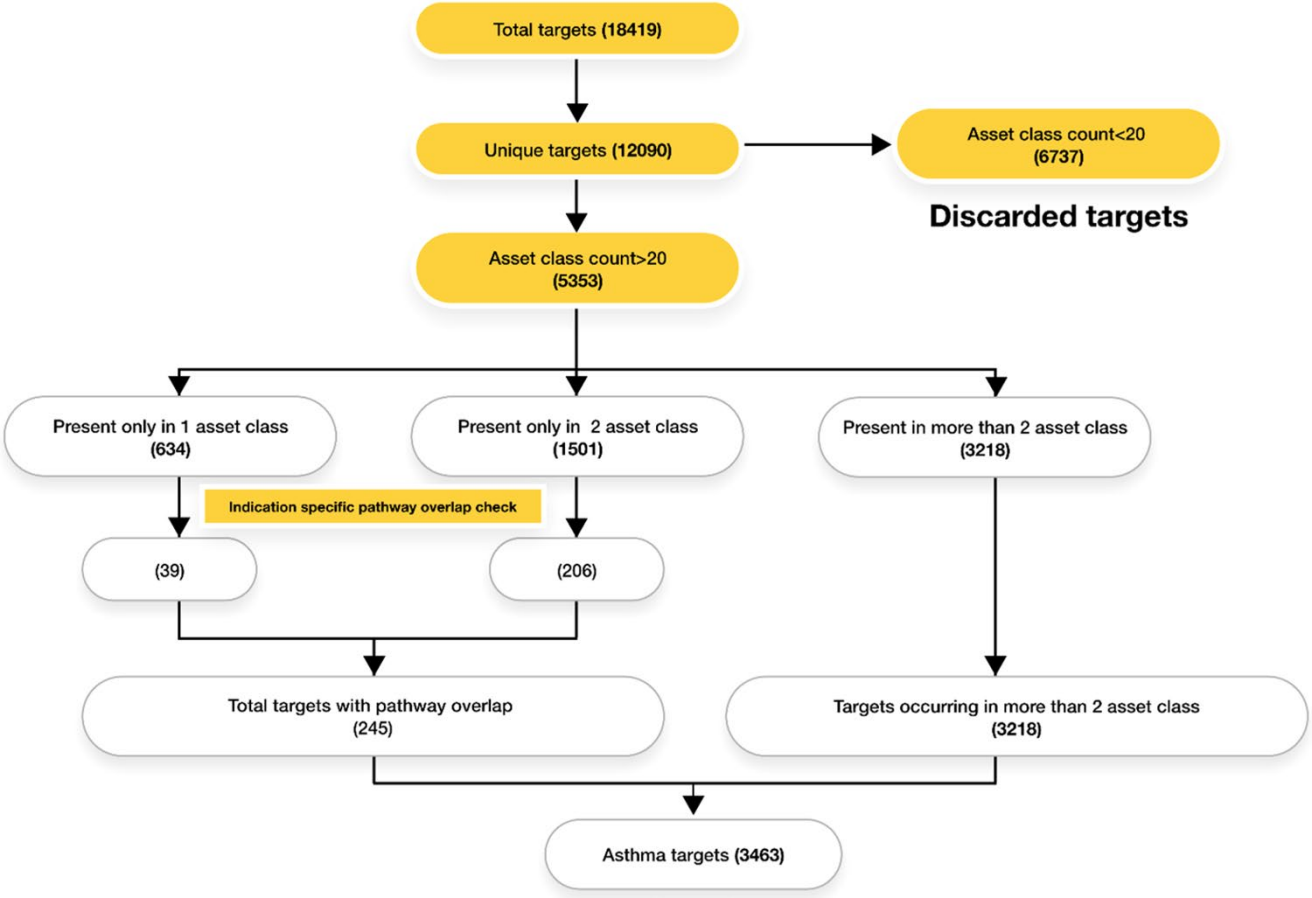
The overall workflow of sourcing and filtering of targets.

This filter reduced the target list to 5353 targets. Targets with an asset class count above 20 but only from one asset class were identified to check the overlap of their pathways with the asthma specific pathways (for disease relevance). Targets that did not have overlap with the asthma specific pathways were excluded from the list. This filter narrowed the target list for asthma down to 3463 targets, which were further used for the evaluation of targets (Fig. 4) (Supplementary File 1).

### Evaluation of identified target based on various biological parameters

#### Evaluation of target druggability

The clinical exploration of a drug which directly or indirectly modulates a protein is a strong indicator of target druggability and this evidence-based approach was followed for exploring the target potential. The likelihood of modulating a target with either a small molecule or an antibody helps define the success probability of a target which makes the druggability parameter an important part of the target assessment pipeline. The drug target relations were extracted from approved drug labels and literature. A target’s small molecule druggability was estimated based on its structural aspects and whether or not a small molecule drug is already approved or under investigation for the target. Targeting proteins using antibodies inside cells has been a long challenging task. The antibody druggability of a target was assessed based on the localization of the target and whether or not an antibody drug is already approved or under investigation for the target. This parameter was evaluated for all targets to understand the druggability for each target protein.

#### Evaluation of proteins to identify potentially unsafe targets

Three groups of potentially unsafe targets were identified using the methodology described by Failli et al.^17^. The described method was modified for the current data and purpose of our analysis. Firstly, targets that had a history of having drugs withdrawn from the market under any jurisdiction were marked as potentially unsafe targets. Targets with drugs that have failed or have been withdrawn from the clinical trials and do not have any successful/ongoing clinical trials in a higher phase were further discarded. Thirdly, genes with functions essential for cellular or organismal viability were discarded as modulation of these genes using a drug may lead to fatal events as referenced by Failli et al.

#### Evaluation of targets for its use as biomarker

Protein targets with biomarker potential have a great significance in drug development. Ontosight^®^ Discover was used to mine scientific documents to extract the targets with biomarker potential. We used the query “biomarker + target name + asthma” as keywords to find relevant hits. Identified targets were further classified into four categories and validated through literature evidence. The biomarkers identified were further classified as diagnostic, prognostic, predictive, or therapeutic.

Definitions used for classification of biomarker:

1. Diagnostic biomarker—A biomarker used to detect or confirm presence of a disease or condition of interest or to identify individuals with a subtype of the disease.
2. Prognostic biomarker—A biomarker used to identify likelihood of a clinical event, disease recurrence or progression in patients who have the disease or medical condition of interest.
3. Predictive biomarker—A biomarker used to identify individuals who are more likely than similar individuals without the biomarker to experience a favorable or unfavorable effect from exposure to a medical product or an environmental agent.
4. Therapeutic biomarker—A biomarker which could become a molecular target for therapy

#### Evaluation of target for asthma associated molecular pathways

The target pathways were characterized by analyzing the involvement of identified targets in the actual pathophysiology of the disease through disease-relevant pathways. The target associated pathways and asthma associated pathways were first extracted from our proprietary database and an overlap of asthma relevant pathways with target associated pathways indicated an involvement of identified targets in asthma pathophysiology through the molecular pathways.

### Evaluation of novel targets

Asthma is an inflammatory disease affecting millions of people including children. The currently available therapies such as β-agonists and glucocorticoids are mainly focused on reducing symptoms in asthmatic patients and do not address the root cause of the disease. Thus, there is a need to discover and identify new drugs for which identification of novel targets is the primary step. A rule-based method was followed to evaluate the targets for novelty based on their discussion in high impact journals (impact factor > 8) in the last 10 years, mentioned in news articles during the last 5 years in association with asthma and no exploration in clinical trials beyond phase 1. The targets satisfying all the three parameters were identified as novel targets and were used to evaluate their role in Asthma.

### Evaluation of target competitive intelligence

We conducted a comprehensive analysis of the clinical trial data from several clinical trial repositories to provide actionable insights related to the shift in the market landscape with respect to the competitors in the market for developing drugs against Asthma, comparative updates related to competing companies and current research in the field of Asthma. Ontosight^®^ Discover was utilized to extract asthma specific clinical trials aggregated from clinical trial registries and were evaluated for the frontrunning sponsor and collaborator companies. The drug target relationship was extracted for the Asthma specific drugs from approved drug labels and literature.

The key insights include:

- The pharma and non-pharma sponsors list funding the clinical trials independently or in collaboration.
- The total number of clinical trials (active, terminated, withdrawn and under recruitment) for each target protein.
- The data related to the total number of drugs tested in the clinical trials for a given target protein. In addition to this, the highest clinical phase for the drugs developed against a particular target.
- Scanning of a particular drug’s mechanism of action (MOA) for assessment of the drug’s pharmacological effect.

### Target prioritization and ranking

Target prioritization was divided into two parts wherein the first part involved percentage-based weightage assignment to the six literature data sources like publications (12%), congress (3%), clinical trials (9%), patents (4%), grants (6%) and news articles (6%). Within the data sources each target was scored based on their recency, impact factor and relevancy in case of publication, recency and position of terms (title, abstract, full text) for grants, congress, news, patents and in case of clinical trials the highest phase, clinical trial status and position of terms (title, abstract, indication, intervention, full text, raw text) were considered. Combining all the six literature data sources, the first part of the prioritization was assigned 40% weightage.

In the second part of the prioritization, disease relevant parameters like druggability (12%), disease-relevant pathways (9%), biomarker potential (3%), target safety (3%), expression and mutation data (12%), indication relevant tissue protein expression (9%) and novelty (12%) were assigned a percentage-based weightage and together these parameters were given a total weightage of 60%.

The final prioritization for each target was based on the cumulative score that was generated after combining the 40% score of the six literature data sources and the 60% score from the disease relevant parameters. The target identification and prioritization pipeline also enable the incorporation of user-input for multi-view interpretation of the same targets. For example, if a user is interested in prioritizing targets based on small molecule druggability, it would be possible to include this input in the prioritization procedure by specifically selecting targets having a known 3D structure and clinical trial data for testing using a small molecule drug.

## Results and discussions

### In silico target evaluation based on various biological parameters

All 3463 targets identified from the target sources as described above in "Combined list of targets" were further subjected to evaluation on the following parameters: druggability, safety, tissue expression, dysregulated markers in asthma conditions, overlapping pathways, known biomarkers and novelty.

#### Evaluation of target for druggability

A total of 2857 targets out of the 3463 targets were found to be druggable either using an antibody or a small molecule from our pipeline. These targets were further categorized into highly druggable (404) or potentially druggable (2453) targets. Highly druggable targets were the targets which had a clinical precedence with a drug against it in clinical trials along with an available 3D structure or surface localization. Potentially druggable targets did not have any clinical precedence but were potentially druggable given the availability of a 3D structure or surface localization. The 404 highly druggable targets were further categorized as small molecule druggable targets (242), antibody druggable targets (68) or both (94). 29 of these 94 targets were identified in the top 100 of our prioritized target lists for asthma (Supplementary File 2).

TNF was one of the top targets identified as highly druggable using small molecule modality as well as antibody modality. TNF is targeted by a number of drugs which have been on the market for a long time although not specifically in asthma. The tumor necrosis factor pathway plays a vital role in immune responses and its dysregulation is implicated in auto-inflammatory diseases including asthma. The increased TNF levels in the body are associated with severe asthma, poorer lung function and worse asthma control^18^. Larger clinical studies failed to show benefit with a heterogeneous response to anti-TNF therapies in asthma^19^. VEGFA was another highly druggable target in the list with clinical precedence, membrane localization and an available 3D structure. Pulmonary diseases like asthma and cystic fibrosis were characterized by the overactivity of the mucus-secreting goblet cells leading to pathologic mucus metaplasia and airway obstruction. Pathways promoting mucus metaplasia involve VEGFA and its receptor KDR which were suppressed leading to increased SOX9^20^. IGF1R (insulin-like growth factor 1 receptor) was also identified as one of the druggable targets and targeting IGF1R ameliorated the typical asthmatic features^21^. PTGS2 (Prostaglandin-Endoperoxide Synthase 2) which regulates the biosynthesis of prostaglandins are important mediators in asthma and are also known to cause eosinophilic inflammation^22,23^ (Supplementary File 2).

#### Evaluation of targets for safety

**Figure 5:**
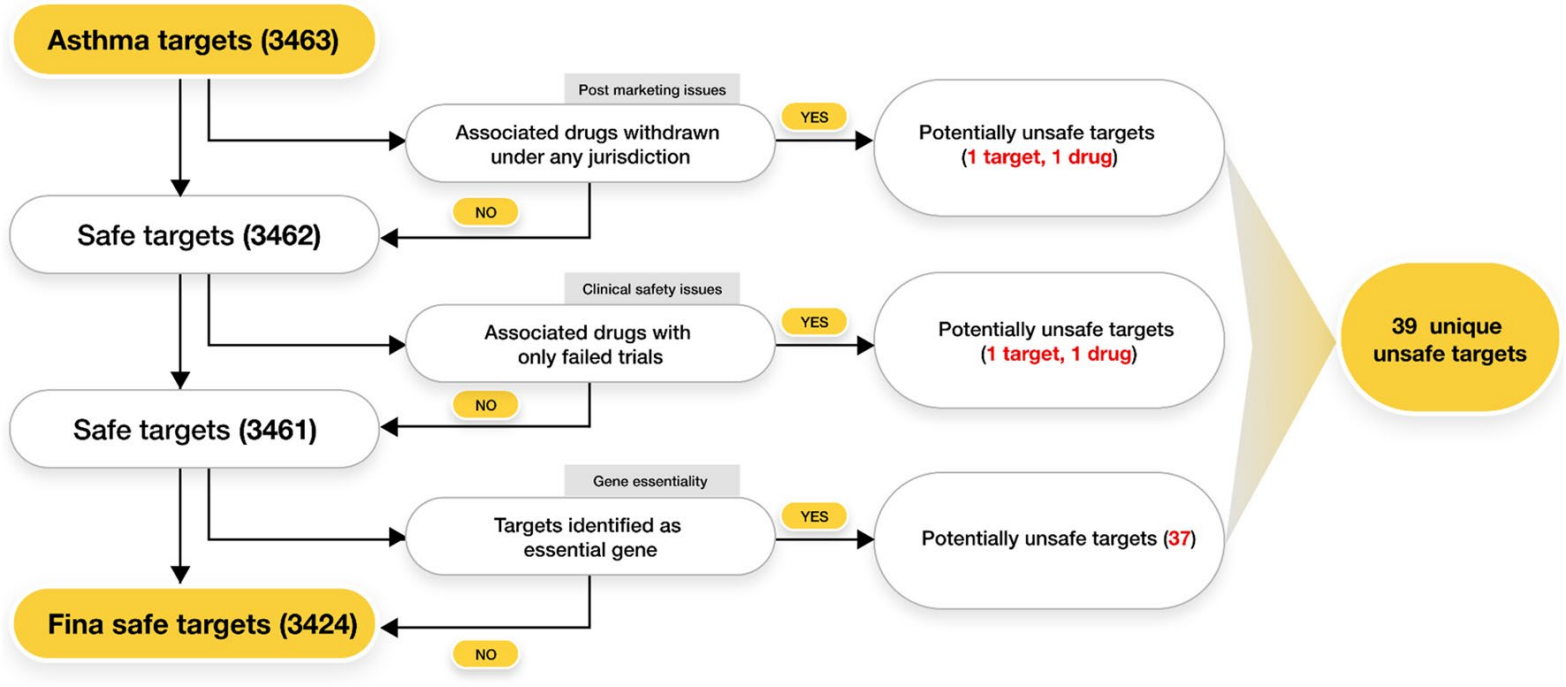
Workflow of identification of potentially safe targets.

In the prioritized list of 3463 targets, three categories for safety were evaluated (Fig. 5). The first category of targets (1) was marked as unsafe due to the withdrawal of their drug from the market under any jurisdiction due to direct reasons associated with the target safety. The second category of targets (1) were the ones whose drugs were withdrawn from the clinical setting due to safety reasons. The third category of targets (37) were the essential genes that were marked as unsafe due to their indispensable role in the biological processes in the body. Together 39 targets were identified and given a low priority based on the safety aspects, in the list of 3463 targets (Supplementary File 3).

Post-marketing issues were identified for SERPINB6, ELN and NID1 where the drugs for these 3 targets were withdrawn from the market. However, ELN and NID1 were removed from this category after further deep dive as the withdrawal reason of the drugs for these two targets were not associated with the targets. Xigris which contained the active substance Drotrecogin alfa was identified as a drug for SERPINB6 from our pipeline which was withdrawn due to the benefit-risk balance issues observed during the annual review by the European Medicines Agency’s (EMA) Committee for Medicinal Products for Human Use (CHMP)^24^.

The two targets marked as unsafe due to withdrawal from clinical setting were APEX1 (Apurinic/Apyrimidinic Endo Deoxyribonuclease 1) and PTPN6 (Protein Tyrosine Phosphatase Non-Receptor Type 6). PTPN6 was later removed from this group as the clinical trial was terminated due to lack of inclusion and was not directly related to PTPN6. APEX1 is known to have a strong role in repair of alkylation and oxidative DNA damage in a cell. Granulocyte colony-stimulating factor and IL-13 cytokines, which are present in asthmatic lungs and during the initiation of fibrosis, enhance AP-1 DNA binding and APEX1 production in normal alveolar macrophages^25^. Our pipeline identified Lucanthone for APEX1 which was found to be terminated in the clinical setting because of the drug’s several adverse events in mass patient testing at higher doses (NCT02014545). There are studies which show that suppression of APEX1 could lead to the accumulation of unrepaired DNA damage in cells^26^.

Essential genes are critical to the survival of the organism and a complete knockout of the gene could pose safety issues. These genes could only be partially modulated and should not affect the essential functions in the organism. INPP5D, PSMB4, PSMD7, USP5, PLK1 are some of the examples of essential genes identified as the third group of unsafe targets from our pipeline which was also evident as these targets are ranked lower in the top 1000 prioritized list of targets. Consider the example of Inositol polyphosphate-5-phosphatase D (INPP5D) which plays an important role in the brain’s defense by building enzymes that help the microglia to engulf damaged brain cells^27^. Proteasome subunit beta type-4 (PSMB4) contributes to the complete assembly of the 20S proteasome complex which may interfere with protein substrate degradation if modulated^28^. These targets are responsible for the maintenance and development of several important functions in the human system which makes them crucial for survival and such modulations must be carried out carefully to avoid any serious events.

#### Evaluation of target for asthma specific tissue expression

Lungs are the primary organ involved in the pathophysiology of asthma. Five targets (IFNGR1, SFTPA1, SFTPA2, C4BPA, SFTPFB) were found to be exclusively expressed in the lungs under normal conditions with 544 targets showing high expression in the lungs along with other organs with varied expression were identified. Surfactant deficiency along with dysregulation of host defense and inflammatory processes were some of the key features in a majority of the pulmonary diseases^29^. HMOX1 (Heme Oxygenase 1) was one of the top targets identified for asthma showing high expression in lungs along with other organs and its induction in a mouse model of ovalbumin-induced eosinophilic asthma suppressed Th2 responses and reduced apoptosis of pulmonary pAECs^30^. IGF1R (Insulin Like Growth Factor 1 Receptor) was also found to be highly expressed in the lungs with other organs and plays an important role in myeloid and airway epithelial homeostasis^31^.

#### Evaluation of targets for genomic analysis (patients raw data)

Identification of genetic markers for asthma disease development and progression is important for better managing the disease and also to develop better drugs. Genomics Expression analysis and mutation analysis helped identify differentially expressed genes (DEGs) in asthma and the genetic variation in the genes which could lead to asthma.

##### Genomics expression analysis

**Figure 6:**
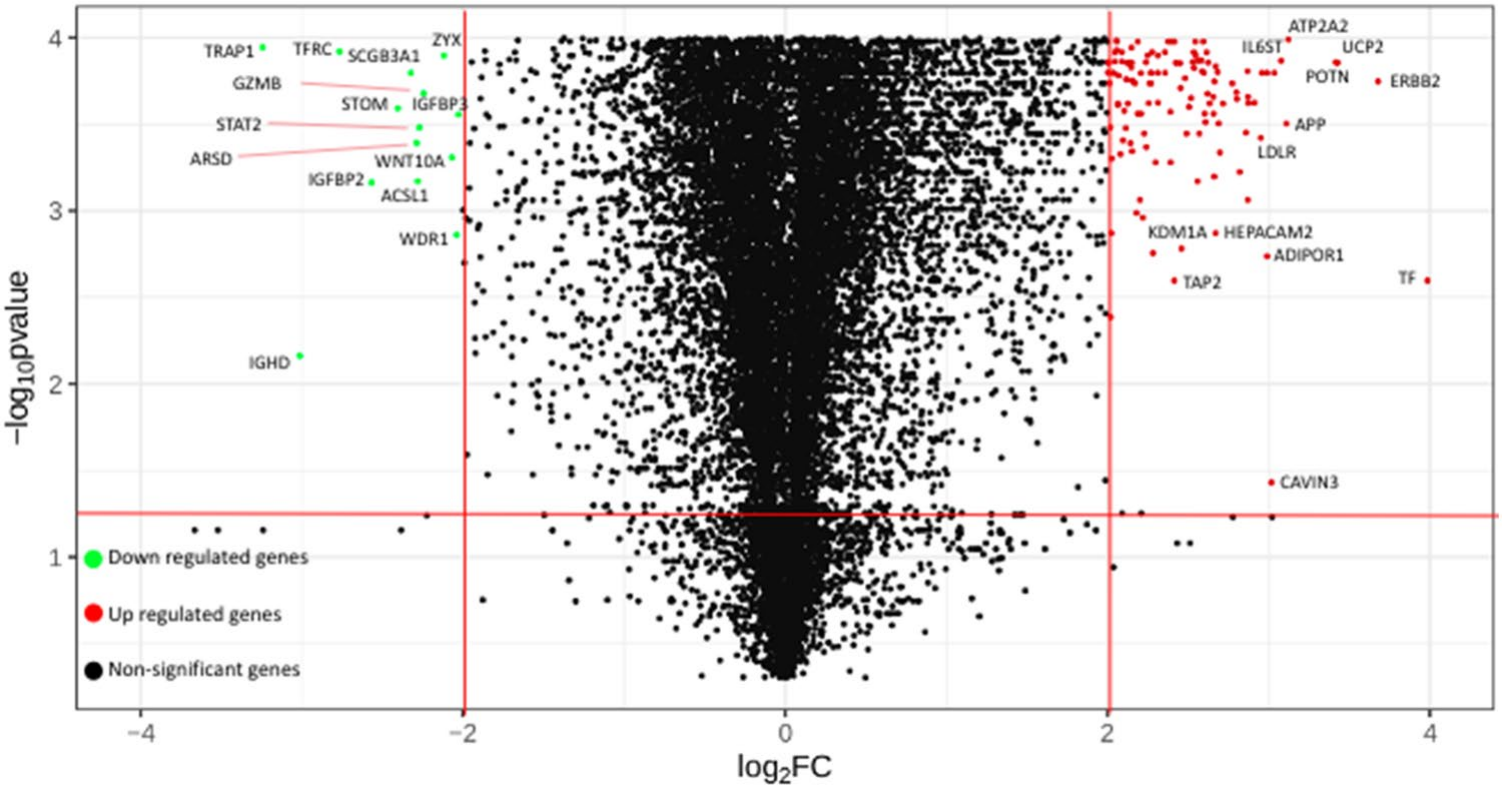
Volcano plot of differentially expressed genes in asthma patients.

A total of 140 unique genes showing significant differential expression in asthmatic patient samples available from GEO (Fig. 6) were identified from the analysis. 123 DEG’s which were identified from genomics expression analysis also showed overlap with literature and variant analysis genes and only 17 DEG’s were uniquely identified from genomics expression analysis. Among these 140 differentially expressed genes, 126 genes were upregulated and 14 genes were downregulated (Supplementary File 4).

The 140 DEGs identified from the genomics pipeline were further evaluated for their role in the asthma pathophysiology based on the pathway enrichment analysis. The top 5 pathways that were found to be associated with the significantly upregulated genes were acyl carrier protein metabolism pathway, signaling by EGFR pathway, cytokine signaling in the immune system pathway, NOTCH signaling pathway and Oncostatin M signaling pathways. EGFR signaling is responsible for mediating airway hyperreactivity and remodeling as reported in many allergic asthma models^31,32^. Allergic asthma is substantially driven by the Th2 immune response, where NOTCH signaling activates the expression of the crucial transcription factor GATA3. Preclinical evidence suggests that inhibiting the NOTCH signaling helps in the reduction of the Asthma phenotype^33^. Oncostatin M signaling pathway is capable of regulating eosinophilic inflammation for airway remodeling that occurs in asthmatic patients. mRNA expression and protein levels of Oncostatin M were reported to be significantly higher in the sputum samples from asthmatic patients.

The top 5 pathways associated with the significantly downregulated genes were PI metabolism pathway, ciliary landscape pathway, membrane trafficking pathway, ferroptosis pathway and innate immune system pathway. In the airways, cilia operate in concert with airway mucus to mediate the critical function of mucociliary clearance, cleansing the airways of inhaled particles and pathogens Downregulation of the targets involved in the ciliary landscape pathway would interfere with the normal clearance and cleansing of the airways^34^. Ferroptosis of adaptive immune cells triggers a series of inflammatory processes with an increase in pro-inflammatory macrophages in asthmatic airways.

##### Genomics mutation analysis

Disease-causative variants were identified by studying single nucleotide polymorphisms (SNPs) that are associated with asthma. Most of the asthma-susceptibility genes that have been identified so far, take part in immune modulatory and inflammatory processes.

The mutation analysis approach could identify overall mutations for 2229 targets combining genomic variant analysis and publicly available structured databases. 1384 targets from the 2229 list were uniquely identified from the variant analysis; these were such targets whose mutation data was not reported in any public databases. Mutations associated with 514 targets were uniquely identified from publicly available structured databases and mutation of 331 targets were identified from both the variant analysis and publicly available structured databases.

Through the genomic mutation analysis, the impact of the variants was measured. Variant impact is the measure of severity of variant consequences. It is classified into three categories

1. High—These variants can have a high impact on protein which may cause protein truncation. Mutation analysis could identify 55 genes which have high impact asthma variants. BRAF (rs145773998), CDK5 (rs145339468), CD38 (rs79840235) were found to have high impact variants in asthmatic conditions.
2. Moderate—Moderate impact variants are non-disruptive variants that might change protein effectiveness. 1604 genes were found to have moderate impact variants through analysis. RELA (rs375768034, rs61759893), ITGAM(rs370936973), ADAM17(rs79932015) genes have been identified with the moderate impact mutations in asthma.
3. Low—Low impact variants are harmless variants and they unlikely change the protein behavior. 257 genes were found to have low impact variants. ANXA1(rs368581314), CD44(rs116243547), BTK(rs150930053) genes have been identified with the low impact mutations in asthma.

**Figure 7:**
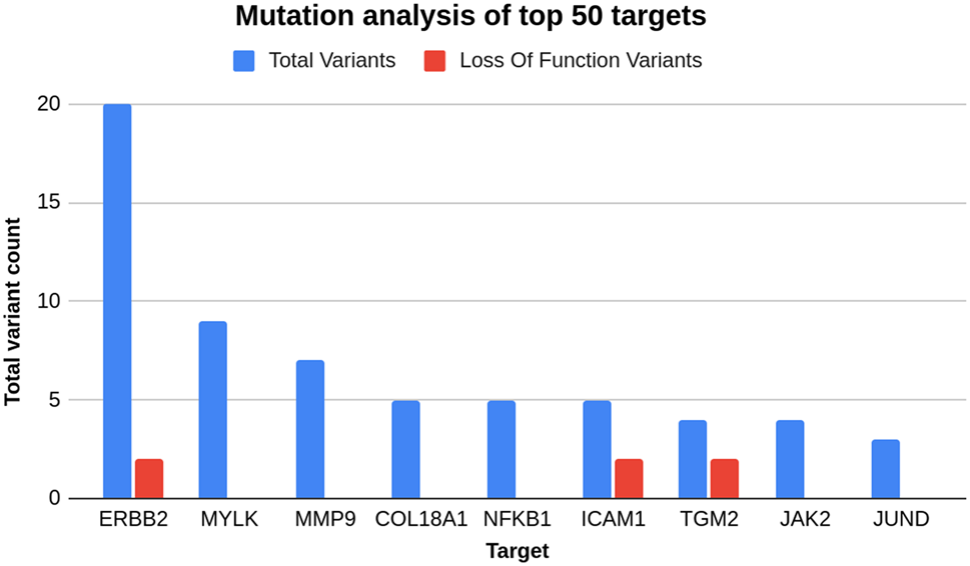
Mutation targets with their variant and loss of function mutation count. Blue bar represents the total number of variants identified from that target and red bar represents the count of loss of function mutations. Out of 9 targets, 3 targets have loss of function mutations.

9 out of top 50 targets were identified to be mutated in asthma patient samples which had come from our pipeline analysis as well as curated databases (Fig. 7). ERBB2, MYLK, MMP9, COL18A1, NFKB1, ICAM1, TGM2, JAK2, JUND were the 9 targets out of which three also had a loss-of-function mutation reported in asthma (Supplementary File 5).

### Mutated targets with loss of function variants

#### Receptor tyrosine-protein kinase erbB-2 (ERBB2)

The expression of ERBB2 was reported to be lower in asthmatic patients^35^ however it plays an important role in delaying wound healing^36^, airway eosinophilic remodeling, inflammation, organogenesis, cellular differentiation and transcriptional modulation^37^. ERBB2 gene mutation is potentially implicated in asthma risk. Pipeline identified 20 single nucleotide polymorphisms (SNPs) and 2 LOF with splice region variant and intron variant (rs1058808, rs1810132, rs2517955, rs2952155, rs2952156, rs1136201, rs35464006, rs61737968, rs200382130, rs1801201, rs150680317, rs55943169). Some of these moderate impact mutations have significant association signals located at the ERBB2 gene, associated with protection for asthma^38^.

#### Intercellular adhesion molecule 1 (ICAM1)

The ICAM1 is the receptor for the major group of rhinoviruses^39^, the most important cause of acute asthma attacks, binding of rhinovirus (RV) to ICAM1 on T-cells may modulate their function^40^. ICAM1 mediated elevated inflammation is implicated in asthma pathogenesis^41^. Pipeline identified 5 single nucleotide polymorphisms (SNPs) and 2 LOF which included missense variants and downstream gene variants (rs5491, rs5498, rs146134321, rs139178890, rs150121537) with moderate impact. These mutations may hamper or activate sites which are essential for rhinovirus induction of ICAM-1 promoter activity in asthma^40^.

#### Transglutaminase 2 (TGM2)

TGM2 was the most differentially expressed in airway cells and airway lining fluid in asthmatic airway epithelium^42^. In-vitro and In-vivo studies confirmed that TGM2 may be a key initiator of inflammatory cascade in asthma conditions^43,44^. Pipeline analysis shows 4 single nucleotide polymorphisms (SNPs) and 2 LOF which includes intron variants, missense variants and downstream gene variants (rs2076380, rs45629036, rs146137365, rs41274720). These all mutations show moderate impact in asthma pathogenesis.

#### Evaluation of targets for biomarker applicability

Asthma management relies on history of symptoms and the bronchial obstruction measurement, for which objectively measurable inflammatory and biochemical processes can serve as surrogate markers. Conventional markers such as blood eosinophils, fraction of exhaled nitric oxide, serum IgE and periostin, feature limited sensitivity and specificity despite their significant correlations^45^. Therefore, there is a great need for identification of robust biomarkers for early diagnosis to track progress of therapy and predict outcome of therapy.

We found a total 92 potentially biomarker targets which were further classified in four categories based on literature evidence. 28 out of 92 were diagnostic biomarkers, 40 were prognostic, 13 therapeutic biomarkers and 7 predictive. 4 targets were categorized into more than 1 group. Interleukin 13 (IL-13), one of the top targets in our prioritized list, was classified under the diagnostic biomarker category. IL-13 is an important cytokine in airway hyper responsiveness, mucus production and various immune reactions. IL-13t can be detected in serum^46^. TNF is proinflammatory cytokine implicated in modulation of inflammation. The clinical response correlated closely with the expression of TNF-α receptor 1 on monocytes. It could be detected in blood, this suggests that TNF measurement might be a useful biomarker^47^. Periostin (POSTN) is ubiquitously expressed in various tissues including lungs and it is involved in many aspects of asthma including eosinophil recruitment, airway remodeling, development of a Th2 phenotype, and also contributes to the increased expression of inflammatory mediators. Periostin levels decrease with inhaled corticosteroid (ICS) therapy and could be detected easily in-patient serum. Based on literature mining it was classified under two categories Diagnostic^48^ and Prognostic^49^. Insulin-like growth factor 1 receptor (IGF1R) was classified as a prognostic biomarker. IGF1R was found to be upregulated in eosinophils of asthmatic patients^50^ and the pharmacological inhibition of IGF1R showed attenuation of bronchial differentiation and goblet cell hyperplasia in house dust mite-induced allergy^31^. All the potential biomarkers from top 100 targets for asthma are mentioned in Table 5.

**Table 5:** Categorization of potential biomarker from top 100 targets for Asthma.

Target	Expression or secretion in	Role in Asthma
Diagnostic biomarkers		
IL13	Serum, Sputum^46^	Induce chemotaxis of eosinophils to the site of injury^51^
TNF	BALF, Blood^46^, Sputum^18^	Pro-inflammatory cytokine and recruitment of neutrophils and eosinophils^52^
ADIPOQ	Plasma, lung^53^	It is a modulator of the innate and acquired immunity response^54^
CCL26	Sputum^55^	Recruits and activates eosinophils in asthmatic patients^55,56^
IL2	Sputum, Serum, EBC^57^	Regulates inflammatory response^57^
HMGB1	Sputum^58^	It plays a central role in eosinophilic airway inflammation in asthma^59^
CCL11	BALF, Blood, EBC and sputum^60^	Play important role in leukocyte migration into the lungs^61^
Prognostic biomarkers		
IL10	Serum^46^	Anti-inflammatory and involved in cytokine activation^62^
IFNG	Serum^63^	IFNG stimulate eosinophil activation, longevity or apoptosis^63^
CRP	Serum^64^	It is associated with Asthma severity and has role in Calcium ion binding^65^
MMP9	BALF, Sputum^66^	MMP-9 play a role in chronic airway inflammation and remodeling^67^
CD14	Serum, Plasma^68^	It is a marker of monocyte/macrophage activation^69^
FGF2	Sputum^70^	Important in tissue development and repair^71^
IL33	Lung^72^	IL-33 plays important roles in type-2 innate immunity^73^
CD86	Blood^74^	Contributes to T lymphocyte activation and expansion^75^
LEP	Serum ^76^	Leptin is elevated in obese individuals, and the risk of asthma^76^
EPX	BW, BAL and serum^77^	It is highly toxic to bacteria and parasites. It regulate inflammation^78^
GC	Sputum^79^	It is important in vitamin D metabolic process and low levels of vitamin D are associated with asthma severity^80^
Therapeutic biomarker		
IGF1R	BAL^81^	Regulating phagocytosis and communication of alveolar macrophages^82^
Diagnostic and prognostic biomarkers		
VEGFA	Serum^83^	Plays an important role in the development of airway remodeling^84^
POSTN	Serum^48^	Eosinophil recruitment, airway remodeling, development of a Th2 phenotype, and contributes to the increased expression of inflammatory mediators^85^

*EBC* Exhaled breath condensate, *BALF* Bronchoalveolar lavage fluid, *BW* Bronchial wash, *BAL* Bronchoalveolar lavage.

### Top asthma targets identified through mining of literature data sources

All the identified targets were sorted based on the combined literature evidence score from 6 different data sources like publications, congresses, grants, news, clinical trials and patents to evaluate and identify the most researched targets from the literature. This helps to generate a baseline understanding of what's known about the pathophysiology of the disease and the key proteins involved. Multiple interleukin targets were identified from all the 6 literature data sources. IL13, IL10, IFNG, TNF, IL18 were the top 5 targets discussed extensively in publications, news articles and congress abstracts related to asthma. These targets were identified to be well-known for their role as pro-inflammatory markers in asthma and their identification through our pipeline increases our confidence in the other targets that were also identified but may not be well-known. A pathways enrichment analysis was further performed on the top 100 targets to understand the role of these targets in the development of asthma. Three major pathways associated with asthma leading to early development and chronicity were enriched with the highest relevance: Cytokine signaling, JAK-STAT signaling pathway and toll-like receptor signaling pathway. Pathways like IL-17 signaling pathway, interleukin-18 signaling pathway also ranked high on the list but were grouped under the Cytokine signaling which plays a pivotal role in asthma initiation and progression pathway. The uniqueness of chronic airway inflammation is that it is intruded by T lymphocytes, eosinophils, macrophages monocytes and mast cells, and occasionally by neutrophils too. Increase in the airway smooth muscle cell thickness along with hypertrophy and hyperplasia are also observed in Asthmatic patients wherein interleukins such as IL4, IL5 and IL13 are type 2 cytokines which facilitate airway eosinophilia, mucus overproduction, bronchial hyperresponsiveness and immunoglobulin E (IgE) synthesis. The overexpression of IL13 is observed through many studies in sputum, bronchial submucosa, peripheral blood, and mast cells in the airway smooth muscle bundle in asthmatics further supporting its role in airway hyperresponsiveness^86^. These pathophysiologically important interleukins were identified as a part of the cytokine signaling in the top 100 list of targets. In the signaling pathways receptor binding of cytokines leads to the activation of members of the JAK kinases. JAK-STAT signaling pathway controls physiologic events that are deregulated in asthma as JAK-STAT pathway involves the membrane to nucleus signaling events, it stimulates the expression of the inflammatory mediators in Asthma. Owing to the active involvement of JAK-STAT pathway, inflammatory targets like IFNG, IL17A were found in the top 100 literature sourced target list which validates the ability of the pipeline to mine and bring out most relevant and frequently discussed targets in the literature sources. A recent study in patients with severe asthma confirmed the significantly elevated airway levels of STAT6 and also identified the major STAT6-expressing cell type in this tissue as the bronchial epithelial cell^87,88^. The targeting of this pathway through inhibition of activating cytokines (IL-4 and IL-13) and their receptors, the JAKs or the STATs, has been shown to have a therapeutic effect on asthma pathology^89^. Multiple evidence suggests that toll-like receptors may be associated with the atypical stimulation of immune responses, contributing to the chronic inflammation seen in asthma^3^.

**Figure 8:**
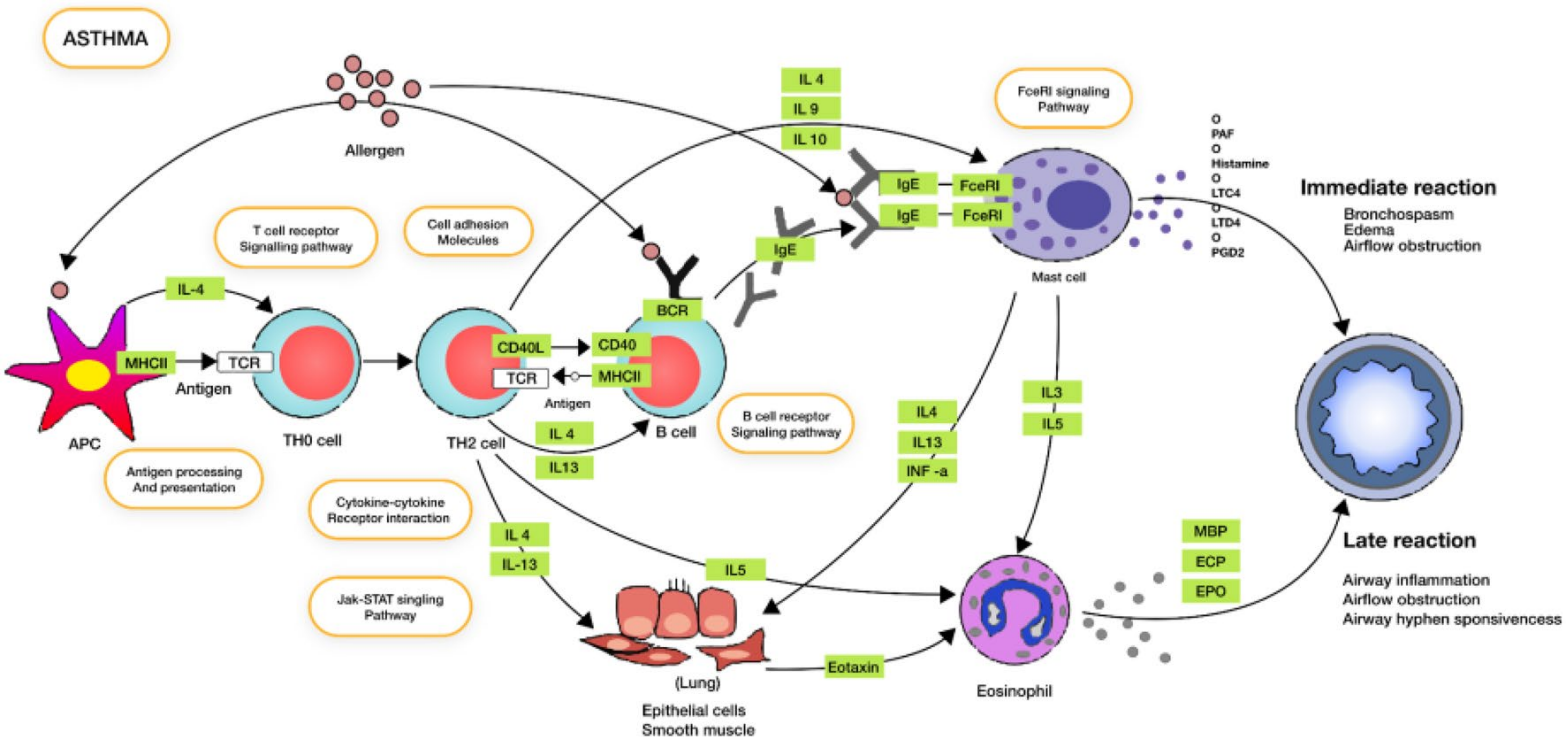
Top pathways associated with asthma. KEGG pathway map of top pathways associated with Asthma: Green blocks represent the top Asthma targets from the prioritized list identified through literature mining of asset classes and their involvement in and inter-connection in various pathways related to Asthma pathophysiology.

We performed a KEGG pathway analysis for the Asthma associated pathways and GO enrichment analysis for the biological processes and molecular functions for the top 100 targets (Fig. 8).

### Competitive clinical intelligence on the targets

**Figure 9:**
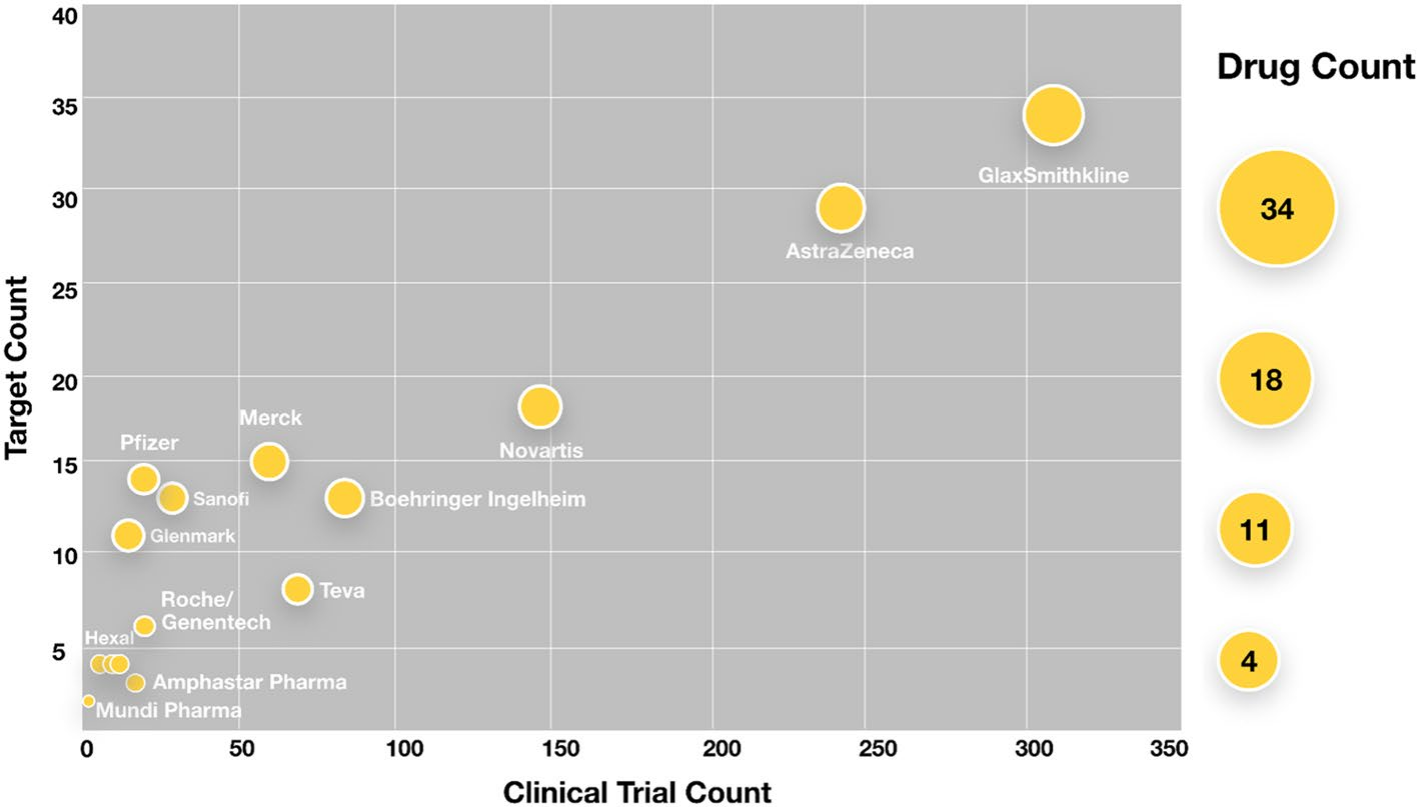
Top sponsor’s in Asthma specific clinical trials.

Ontosight^®^ Discover was utilized to extract clinical trial data from multiple registries to analyze trials across the globe. Based on this analysis our pipeline has identified 2942 clinical trials that are directly associated with 93 targets who have reached or completed the clinical stage testing for asthma. GlaxoSmithKline, AstraZeneca and Novartis were identified as the top 3 front-runner companies actively working to develop drugs for asthma (Fig. 9).

GlaxoSmithKline was identified as one of the top companies rigorously exploring therapeutic assets (60 drugs) for the treatment of Asthma in 312 clinical stage trials. Some of the top targets that the company has eyed over the years are ADRB2, PLA2G4A, NR3C1. ADRB2 (β2-adrenergic receptor gene) is known to modify response to therapy in Asthma patients^90^. Salbutamol, Levosalbutamol, Formoterol, Arformoterol were the top drugs identified for ADRB2, all of which are approved, that were explored in 102 clinical trials for Asthma. Current Asthma treatment helps to prevent asthma attacks and relieve symptoms when exacerbations occur, studies related to the Asthma pathogenesis have led to the development of biologics targeting the cytokines as cytokines have a major role in Asthma. Cytokines like IL5, IL4, IL13 are important inflammatory targets in Asthma, on similar lines GlaxoSmithKline has tapped into the field of biologics where they are exploring their asset Depemokimab which is an IL5 inhibitor in the phase 3 clinical trials. Being the primary cytokine IL5 is involved in the survival of eosinophils, inhibition of this pathway would help to reduce eosinophilic airway inflammation. Depemokimab is one such long acting IL5 inhibitor and the clinical trial is scheduled to be finished in late 2023 and if approved by FDA, this drug would add to the already approved IL5 antibody drugs in the GlaxoSmithKline pipeline.

AstraZeneca was the second top pharmaceutical in the list of competing companies for drug development in Asthma leading 242 clinical trials and exploring the potential of 53 drugs in total for Asthma at different time points. The 53 drugs primarily focused on modulating 29 targets. IL33 is one such target explored by AstraZeneca in the phase 2 clinical setting for Asthma. IL33 being an important cytokine is a key regulator of immune response and tissue remodeling in chronic inflammatory diseases wherein it interacts with different set of receptor proteins that form surfaces such as the linings of the airways in our lungs, this way tissue remodeling changes how cells organize and specialize to perform different roles. The company has developed a biological drug Tozorakimab which is an IL33 inhibitor for Asthma treatment (NCT04570657). AstraZeneca was also found to be exploring other interleukins like IL4Ra and exploring its therapeutic modulation using AZD1402 inhibitor in the phase 2 clinical trial for Asthma (NCT04643158, NCT03574805).

Novartis was identified as the third leading company exploring and actively testing assets in Asthmatic patients in the clinical setting. ADRB2 variants has been reported to be associated with airway hypersensitivity, asthma severity, and the response to medications (PMID: 19905915), this target has been explored by Novartis where it has developed drugs like QVM149 Indacaterol, Salmeterol, Formoterol, Arformoterol Levosalbutamol. QVM149 is an inhaled treatment under development for patients whose lives remain impacted by asthma despite current treatment with LABA/ICS. In 2020, QVM149 was approved for use in asthma patients in the EU and Japan, where the therapy is marketed as Enerzair Breezhaler. Modulation of interleukins like IL17A is also being explored by Novartis drugs like CJM112 which is a human immunoglobulin G1 (IgG1) monoclonal antibody with potential anti-inflammatory activity. IL-17A is upregulated in inflammatory diseases and plays a key role in the development of inflammation and the immune response.

The competitive landscape from a bird's-eye view shows that the most known targets like ADRB2, PLA2G4A, NR3C1 which are already known inflammatory targets in Asthma are well explored by all the top companies in Asthmatic patients. Target class like interleukins are some of the upcoming targets that are being explored in the recent years where all the top companies in the field of Asthma like GlaxoSmithKline, AstraZeneca, Novartis are shifting their line of experiments from the traditional targets to other potential set of new targets like IL33, IL17A, IL5, IL4, IL13 who have strong association with Asthma pathophysiology and might show promising treatment results in the future (Supplementary File 6).

### Identification of novel targets

We did a reverse screening to identify the novel targets for Asthma based on certain criteria that relate to clinical trials and literature evidence. Our pipeline could identify 121 novel targets from the total list of 3463 targets identified for Asthma. The top novel targets in the list were majorly identified from the unstructured database through literature mining based on the criteria discussed in the method section. Based on the data available for the targets, our algorithm classified the novel targets into three categories of “known knowns”, “known unknowns” and “unknown knowns”. The “known knowns” are the targets which are frequently discussed with relation to Asthma and their role in the pathophysiology of the disease is known but their therapeutic feasibility is not yet explored. The “known unknowns'' are the class of novel targets which are known to be involved in the disease but the exact mechanism leading to their contribution in diseased condition is not known. We identified these targets from literature mining where there could be targets for which the mechanistic details are still being explored. The next category of “unknown knowns” are the novel targets which have not been identified yet but may be associated with the pathophysiology of the disease by known mechanisms. We identified these targets through our genomics analysis pipeline. Additionally, we deploy certain parameters wherein our algorithm assigns these novel targets a score based on the extent of their clinical exploratory data available and the discussion of the targets with respect to Asthma in high impact factor journals which helps to prioritize the novel targets (Table 6).

**Table 6:** Top novel targets and its association with asthma.

Target	Role in asthma
POSTN	Periostin is associated with pathogenesis of asthma-associated inflammation in asthma^91^. Periostin is elevated and found to be a biomarker of type 2 inflammation^92^. POSTN expression upregulated by IL13 and IL4 cells which has been reduced by treatment with anti–IL-13 (lebrikizumab, tralokinumab) Serum Periostin could be utilized as biomarker for the airway wall thickness in Asthma^93^
COL18A1	COL18A1 encodes the alpha chain of type XVIII collagen and is the most abundant airway extracellular matrix component, primary determinant of mechanical airway properties^94^. Epigenetic association studies have shown COL18A1 is associated with lung function development through DNA methylation^95^
JUND	Most of the inflammatory and immune genes contain binding sites for activator protein (AP-1) which is an array of dimeric basic region-leucine zipper proteins of Jun (c-Jun, JunB, and JunD) and Fos (c-Fos, FosB, Fra1, and Fra2) subfamilies^96^. Recent animal studies indicate that the anti–c-Jun, anti-JunD, and anti–c-Fos antibodies were all able to partly remove the AP-1 complexes and this AP-1 inhibition in the airways may have therapeutic value in the control of established asthma^97^
CCR7	CC chemokine receptor 7 (CCR7) is directly involved in the pathogenesis of DC‑ and T cell‑mediated allergic asthma^98^. It plays an important role in the development of ASM hyperplasia in asthma. CCR7 CD4 cells of patients showed significant clinical implications in atopic asthma^99^
ADIPOQ	ADIPOQ gene responsible for expression and secretion of adiponectin which is also associated with the obesity-associated asthma phenotypes^100^. It is an anti-inflammatory adipokine that increases insulin sensitivity and has cardiovascular protection actions. It is a modulator of the innate and acquired immunity response in asthma^101^
PTEN	Phosphatase and tensin homologue (PTEN) block the action of PI3K by dephosphorylating the signaling lipid phosphatidylinositol 3,4,5-triphosphate. There were supportive interactions of PI3K-Akt-mTOR and STAT3-miR21-PTEN which controls IgE induced airway remodeling in allergic asthma^102^. In animal studies there is remarkable reduction in bronchial inflammation and airway hyperresponsiveness observed by intratracheal administration AdPTEN. The above findings indicate a pivotal role of PTEN in asthma^103^
CRP	The high C reactive protein concentration and the age has been found to be associated with the risk of asthma development^104^. The raised levels of high sensitivity CRP are significantly associated with respiratory state of asthma exacerbation and allergic inflammation^105^
IL17RB	IL17RB is a cognate receptor of IL25 and activation of IL-17RB amplifies allergic-type inflammatory responses by promoting Jun kinase (or JNK), p38 mitogen-activated protein kinase (or MAPK), and nuclear factor-kappaB^106^. IL-17RB + granulocytes from peripheral blood were increased in subjects with asthma^107^
ADAR	Adenosine is a potent bronchoconstrictor with either pro- or anti-inflammatory effects depending on receptor interactions^108^. ADAR1 expression levels and protein activity could promote the progression of Asthma. Genetic polymorphisms of adenosine receptors A(1) and A(2A)have been found to be associated with aspirin-intolerant asthma^109^
TGM2	Transglutaminase 2 (TGM2)TGM2, a novel mediator of asthma pathogenesis, is overexpressed in asthmatic airways^44^. TGM2 was involved in mediating the increased cough frequency in EB through the regulation of TRPA1 and TRPV1 expression^43^

Heme oxygenase-1 (HO-1), HMOX1 functions in heme catabolism, cytoprotection and reducing inflammation. HMOX1 was identified as a potentially druggable target through our pipeline based on its PDB structure availability and membrane localization which makes it more easily accessible for any small molecule or antibody drug development. It has a role in cytokine signaling, interleukin pathway and is highly expressed in response to various stimuli related to cellular stress and reactive oxygen species (ROS), cytokines, inflammatory mediators, and infection^110^. Our pipeline was able to identify a variant (rs2071747) for HMOX1 which is associated with Asthma. High linkage disequilibria between the HMOX1 single nucleotide polymorphism (SNPs) and the GT repeat polymorphism has been previously reported to be associated with emphysema^111^. From preclinical studies, it is clear that HO-1 activity may be clinically useful in the management of asthma^112^.

Integrin alpha-M (ITGAM) affects airway smooth muscle (ASM) cell proliferation and viability in asthma. ITGAM showed medium level of expression in lungs and can act as biomarkers of inflammation in Asthma. 2 variants of ITGAM were identified to have moderate and modifier effects in Asthma through our genomics analysis. Although there is no strong clinical precedence for ITGAM, based on our druggability criteria of structure availability and membrane localization, we could categorize this target as potentially druggable using either a small molecule or an antibody drug. Various integrins coordinate to mediate the movement of eosinophil in the airways of Asthma patients which was evident in the GO enrichment analysis. All the evidence directs towards ITGAM being a potentially promising therapeutic target for Asthma.

DExD/H-box helicase 58 (DDX58, also known as RIG-I) is a protein involved in viral double-stranded RNA recognition and type-I IFN production and was originally described as a key mediator of antiviral and innate immune responses^113^. It has been reported that autophagy mediates the degradation of DDX58^114^. DDX58 is co-expressed with BPIFA1, which plays a key role in the regulation of airway surface liquid volume and serves in host defense against bacterial infection in asthma^115^. Rhinoviruses induced upregulation of DDX58 (RIG-I) was enhanced in asthma compared to control^116^. Polymorphism in DDX58 shows significant associations with asthma attacks in the Copenhagen Prospective Study on Asthma in Childhood (COPSAC) study^117^.

Surfactant Protein D (SFTPD), In the lungs, clearance of infectious agents and regulation of inflammatory responses are important for first-line defense, where surfactants play a role in host defense mechanisms. Pulmonary surfactant associated protein D is a multimeric collection that is involved in innate immune defense and expressed in pulmonary, as well as non-pulmonary epithelia^118^. SFTPD directly binds to the eosinophil surface, leading to inhibition of extracellular trap formation and reduction in airway inflammation^119^. It is involved in the toll-like receptor signaling pathway which is one of the primary pathways involved in Asthma. Mutation data from structured sources showed the association of the rs721917 variant with Asthma. According to literature reported data, rs721917 has been shown to be associated with multiple respiratory diseases and is associated with 39% of the variation in SFTPD^120^.

A disintegrin and metalloprotease 17 (ADAM17) is a membrane-anchored proteinase that is the major reason for its multifunctionality and its high similarity with other metalloproteases. The expression in human lung tissue and its expression is upregulated in lung conditions including asthma. Our algorithm has identified 6 SNPs belonging to ADAM17 which can cause asthma. There is some evidence which suggests the correlation between ADAM17 SNPs and change in its expression in asthmatic conditions^120,121^. The GO enrichment analysis showed that integrin binding of ADAM17 is an important molecular function involved in cytokine signaling, signaling by Interleukins pathway, JAK-STAT signaling pathway, GPCR signaling, toll-like receptor signaling pathway. Various small molecules and antibodies have been developed against ADAM17 using different approaches for cancer and inflammation, but none of the drug has reached clinical trials due to two major reasons: its multifunctionality and its high similarity with other metalloproteases. To overcome these limitations, several approaches have been utilized to develop molecules able to discriminate between ADAM17 and its relatives, and to inhibit ADAM17 in a specific tissue or cell-type. Thus, there is still scope for further exploration of ADAM17 as a potential novel therapeutic target which has been demonstrated from the analysis and supported by literature review.

These are some top targets identified from the pipeline which show strong association with respect to the pathophysiology of Asthma however not extensively explored and tested for therapeutic benefit. We propose that these targets could bring therapeutic benefits in Asthma treatment in the upcoming times.

### Target prioritization

The algorithm for target identification is an automated workflow that enables the integration of literature-identified targets using the proprietary Innoplexus' platform with other disease relevant parameters that define druggability, involvement in disease pathology, safety, biomarker potential, genomic and variant data, clinical precedence, novelty and competitive market for the targets. In total we identified 3463 targets for asthma prioritized based on a combined score (Supplementary File 7).

The pipeline was able to identify the most well-known and disease relevant targets in the top prioritized list which validates the rationale used to develop the pipeline and the outputs generated by it. Some well-known targets like Interleukin 13 (IL13), Tumor necrosis factor (TNF), Vascular endothelial growth factor A (VEGFA) were present in the top 10 targets identified for Asthma. Based on the asset class screening IL-13 was identified as the most discussed target for Asthma in terms of its role in the disease pathophysiology in publications, news, congresses, grants and clinical trials. IL-13 is an important inducer of fibrosis in numerous seditious and autoimmune conditions. A major function of IL-13 in the asthmatic airway is to induce chemotaxis of eosinophils to the point of injury. The pipeline was able to identify and evaluate the therapeutic potential of IL-13 drugs that have been extensively tested in the clinical setting for asthma where safety and efficacy data have shown to have no serious adverse events against this target which has provided the path to leading companies like Roche, Genentech and AstraZeneca for developing drugs against IL-13 to treat asthma. TNF was identified as the second top target from the pipeline that holds strong potential for therapeutic benefit in Asthma. TNF is a proinflammatory cytokine that plays an important role in airway diseases. Based on the overlapping pathway analysis it was found that TNF takes part in several inflammatory pathways that contribute to the pathogenesis of Asthma which included the Cytokine signaling, Toll-like receptor signaling pathway and JAK-STAT pathways. From the druggability standpoint, TNF is feasible to target using an antibody as well as a small molecule drug. Adalimumab and Golimumab were the two drugs identified through the pipeline that have been used in the treatment of asthma when patients do not respond effectively to inhaled glucocorticoid therapy. VEGFA is another important cytokine that contributes to the increase in the vascular permeability at the site of the inflammation. Multiple drugs have been identified through the pipeline targeting VEGFA in asthma as an add-on steroid therapy to control the disease. One such drug identified is Minocycline which is under evaluation in the clinical setting for its anti-inflammatory properties in the treatment of Asthma. The pipeline was able to identify 6 different variants of VEGFA which could be associated with the abnormal lung functioning in Asthma. VEGFA has also been explored for its biomarker properties in Asthma. The pipeline identified several articles through the literature mining that directs towards VEGFA being a strong biomarker candidate with a high specificity^122^.

Apart from identifying and prioritizing the most extensively studied targets in Asthma, the pipeline is able to bring out novel targets which hold strong potential in the emerging mechanistic insights associated with asthma which are currently not well explored. These novel targets identified through the pipeline can be effective alternatives in the future as compared to the mainstay therapies that have not shown therapeutically promising results in Asthma.

### Supplementary Information

Supplementary Information 1.

Supplementary Information 2.

Supplementary Information 3.

Supplementary Information 4.

Supplementary Information 5.

Supplementary Information 6.

Supplementary Information 7.

## Data Availability

All data generated or analyses during this study are included in this published article [and its supplementary information files].
